# Clinical phenotypes and disease outcomes of patients with systemic lupus erythematosus meeting indication for primary aldosteronism testing

**DOI:** 10.1177/09612033261473968

**Published:** 2026-07-31

**Authors:** Matthew Wong, Muhammad Akram, Joanna R. Kent, Peter J. Fuller, Alberta Y. Hoi, Jun Yang, Fabien B. Vincent

**Affiliations:** 1 Department of Medicine, 2541 Monash University, Clayton, VIC, Australia; 2 Centre for Endocrinology and Reproductive Health, 518514 Hudson Institute of Medical Research, Clayton, VIC, Australia; 3 Department of Nephrology, 2538 Monash Health, Clayton, VIC, Australia; 4 Department of Endocrinology, 2538 Monash Health, Clayton, VIC, Australia; 5 Department of Rheumatology, 2538 Monash Health, Clayton, VIC, Australia; 6 Centre for Inflammatory Diseases, Department of Medicine, School of Clinical Sciences at Monash Health, 2541 Monash University, Clayton, VIC, Australia

**Keywords:** aldosterone, cardiovascular, hypertension, primary aldosteronism (PA), renin, systemic lupus erythematosus (SLE)

## Abstract

**Background:**

Hypertension is prevalent and contributes significantly to cardiovascular risk in patients with systemic lupus erythematosus (SLE). Primary aldosteronism (PA), the most common endocrine cause of hypertension, confers higher cardiovascular risk than essential hypertension and has been linked to autoimmune diseases including SLE. While the prevalence of PA in SLE is unknown, a third of patients in a tertiary lupus clinic have been demonstrated to have clinical indications for PA testing.

**Objectives:**

To compare SLE phenotypes and outcomes in patients attending the clinic with and without indications for PA testing.

**Methods:**

In this retrospective study, hypertension was defined as blood pressure ≥140/90 on two clinic visits, or a documented diagnosis of hypertension. Baseline demographic and SLE disease characteristics were compared between patients with and without an indication for PA testing as determined according to the 2016 Endocrine Society guidelines, before multivariable regression analyses assessed for associations between having an indication for PA testing with disease phenotypes and outcomes specifically within the subset of patients with hypertension.

**Results:**

Of 322 patients with SLE, 95 patients (30%) had at least one indication for PA testing. These patients were older, had longer SLE disease duration, higher body mass index, higher blood pressure, and more baseline organ damage compared to those without an indication for PA testing. Over the period of clinic attendance (median 6.8 years), these patients had greater accrual of organ damage, higher flare rates, and more frequent proteinuria. Within the subset of hypertensive patients, indications for PA testing were independently associated with reduced baseline renal function (adjusted OR 3.29, 95% CI 1.30–9.47*; p* = 0.017), higher baseline organ damage (adjusted OR 1.94, 95% CI 1.03–3.73*; p* = 0.043), and higher prevalence of flare throughout the study period (adjusted OR 2.86, 95% CI 1.04–8.60; *p* = 0.049) in multivariable modelling.

**Conclusion:**

SLE patients with an indication for PA testing exhibit a phenotype characterised by impaired renal function, increased organ damage and higher prevalence of flare. Further research is needed to prospectively assess whether the increased burden is attributable to PA, other causes of hypertension, or the severe hypertension.

## Introduction

Systemic lupus erythematosus (SLE) is a systemic autoimmune disease, predominantly affecting women of child-bearing age.^1^ Cardiovascular disease is a leading cause of mortality in SLE, with myocardial infarction and ischemic stroke occurring at twice the rate of the general population.^2^ Hypertension, a key risk factor for cardiovascular disease, has been reported in multiple cohorts of SLE patients to have a prevalence of over 50%,^3,4,5,6^ with drug-resistant hypertension also found to be twice as prevalent in individuals with SLE compared to controls without autoimmune disease.^7^

Factors such as renal impairment from lupus nephritis, glucocorticoid treatment, and endothelial damage from chronic inflammation have been suggested to play a role in the pathophysiology of hypertension in SLE.^8^ Another implicated mechanism is disruption of the renin-angiotensin-aldosterone system.^8^ In particular, primary aldosteronism (PA), the most common endocrine cause of hypertension characterised by autonomous aldosterone excess, may be associated with a greater risk of new onset of autoimmune diseases, including SLE, compared to essential hypertension.^9^ Despite PA conferring a higher risk of cardiovascular mortality compared to essential hypertension,^10,11^ and being highly treatable and potentially curable,^12^ PA remains significantly underdiagnosed.^13,14,15^ In a recently published study, we reported that 30% of a cohort of 322 patients with SLE had an indication for PA testing but only 11% of these patients were screened.^16^ In this follow up study, we sought to assess the same cohort for differences in SLE phenotypes and clinical outcomes between patients who had an indication for PA testing and those who did not, with the hypothesis that having an indication for PA testing would be associated with poorer outcomes, particularly cardiovascular and renal.

## Materials and methods

### Study setting and participants

As previously reported,^16^ we identified a cohort of 322 patients who fulfilled the classification criteria for SLE (1997 American College of Rheumatology revised criteria^17^ or the Systemic Lupus International Collaborating Clinic criteria^18^) and had attended the Monash Health (Melbourne, Australia) Lupus Clinic from January 2013 to October 2022. Among them, 95 patients had an indication for PA testing, while 227 did not. These indications were determined according to the Endocrine Society 2016 guidelines and included marked hypertension with blood pressure ≥150/100 mmHg on three separate occasions, drug-resistant hypertension, hypertension with concurrent hypokalaemia or obstructive sleep apnoea, or hypertension with family history of early onset stroke.^12^ The inclusion and exclusion criteria for this cohort have previously been described.^16^ This study was approved by the Monash Health Human Research Ethics Committee (Ref: 89586/MonH-2022-336383).

### Data collection and clinical assessment

Data were retrieved from the Australian Lupus Registry and Biobank (ALRB) database^19^; these data included socio-demographic and phenotypic data prospectively recorded at recruitment and/or routine and annual clinic visits, including activity of the disease, presence of flare and organ damage, routine laboratory tests and medication use. For each patient, the study period was defined as the time between the first visit from which blood pressure was recorded (i.e. baseline) up to the most recent visit during the period of data collection. Disease duration was defined as the time from the date of diagnosis of SLE by a specialist to the date of the visit where data were analysed. Disease activity was assessed using the SLE Disease Activity Index 2000 (SLEDAI-2K),^20^ with active disease defined as SLEDAI-2K >4.^21^ A patient was deemed to have organ-specific active disease in the presence of a positive score in at least one component of the SLEDAI-2K pertaining to the specific organ domain.^21^ High Disease Activity Status (HDAS) was defined as SLEDAI-2K ≥10.^22^ Average disease activity across the study period was calculated using the Adjusted Mean SLEDAI-2K (AMS) score,^23^ which evaluates the mean SLEDAI-2K score adjusted for irregular time intervals between visits.^21^ Glucocorticoid use was calculated using cumulative dose of prednisolone throughout the study period as well as adjusted mean prednisolone dose, also adjusted for time intervals between visits. Flare was assessed using the Safety of Estrogens in Lupus Erythematosus National Assessment (SELENA) Flare Index (SFI) and classified as mild/moderate or severe flares.^24^ Annual average flare rate was calculated from the number of flares recorded throughout the study period divided by the study duration in years. Organ damage was assessed at baseline and annually using the SLICC/ACR Damage Index (SDI),^25^ where presence of organ damage was defined as SDI >0. Accrual of organ damage was defined as an increase in SDI score from baseline to last recorded visit. Reduced eGFR was defined as eGFR below 60 ml/min/1.73 m^2^. The use of immunosuppressants was defined as the use of any of the following medications: azathioprine, cyclosporine, cyclophosphamide, leflunomide, mercaptopurine, methotrexate, mycophenolate, sulfasalazine, tacrolimus, abatacept, adalimumab, baracitinib, belimumab, certolizumab, etanercept, infliximab, rituximab, tocilizumab or tofacitinib.

### Statistical analysis

Data analyses were performed using GraphPad Prism (Version 9.5.1) and R (Version 4.5.2).^26^ Data were presented as mean (standard deviation (SD)) for normally distributed continuous variables, median (interquartile range, Q1-Q3) for non-normally distributed continuous variables, or as counts and percentages (n (%)) for categorical variables. The differences between groups were assessed using *t*-test for continuous normal variables, and Mann-Whitney *U* test for non-normal variables. For categorical variables, Fisher’s exact test was used to compare groups. The Shapiro-Wilk test alongside visual inspection via histogram was used to assess normal distribution. 

Univariable and multivariable regression analyses were performed within the subset of SLE patients with HTN to assess associations between having an indication for PA testing (exposure) with disease phenotypes and clinical outcomes. Covariates adjusted for in multivariable modelling were selected a priori based on clinical relevance and included age, sex, self-reported ethnicity, disease duration, and use of glucocorticoids, hydroxychloroquine, and immunosuppressants; they were first evaluated with univariable analysis for association with each outcome of interest, with significant covariates using a *p*-value cut-off of <0.1 then included in the final outcome-specific multivariable model. Logistic regression was fitted for binary outcome variables, with effect size presented as adjusted odds ratio. Linear regression was fitted for continuous outcome variables with effect size presented as estimated coefficient (β); where continuous outcome variables were skewed, they were log-transformed with a small adjustment factor of 0.1 added to outcome variables to enable log-transformation prior to fitting the linear model, and coefficients were exponentiated and interpreted as ratio of odds. Count outcome variables characterised by over-dispersion were analysed using negative binomial regression with effect size reported as incidence rate ratio (IRR). A two-sided *p*-value <0.05 was considered statistically significant.

## Results

We compared baseline demographics (Table 1), and SLE disease characteristics (Table 2), between patients who had at least one indication for PA testing during the study period (*n* = 95), and those who did not (*n* = 227). At baseline, patients with an indication were significantly older (47.7 [IQR 37.1–61.3] vs 39.5 [IQR 30.1–49.3] years, respectively; *p* < 0.01) and had longer disease duration (8.5 [IQR 3.5–18.1] vs 5.6 [IQR 1.0–13.3] years, respectively; *p* < 0.01), as well as had a higher body mass index (26.1 [IQR 22.4–31.1] vs 22.9 [IQR 20.6 –25.9] kg/m^2^, respectively; *p* < 0.01). The group with an indication for PA testing also had a significantly higher proportion of patients of European ethnicity and lower proportion of patients of Asian ethnicity compared to the group without an indication. As expected, patients with an indication for PA testing also had higher systolic blood pressure (135.3 ± 14.2 vs 119.3 ± 12.2 mmHg respectively; *p* < 0.01) with a higher proportion taking at least one antihypertensive medication (56% vs 31%, *p* < 0.01) at baseline. Significantly more patients in the group with an indication for PA testing had organ damage (66% vs 41%; *p* < 0.01), including both cardiovascular (15% vs 6%; *p* = 0.01) and renal (18% vs 5%; *p* < 0.01) damage, as well as reduced eGFR (22% vs 4%; *p* < 0.01). Of note, no significant differences were identified in disease activity, presence of proteinuria, flare, and medication use including hydroxychloroquine and glucocorticoid exposure, between those two groups at baseline.

**Table 1.** Baseline demographics and non-SLE specific clinical characteristics in patients stratified by indication for PA testing.

​	PA testing		*p*-value
	Indication (*n* = 95)	No indication (*n* = 227)	
Age (years), *median (IQR)*	47.7 (37.1–61.3)^a^	39.5 (30.1–49.3)	<0.01
Female sex, *n (%)*	78 (82%)	201 (89%)	0.15
Body mass index, *median (IQR)*	26.1 (22.4–31.1)^b^	22.9 (20.6–25.9)^c^	<0.01
Smoking status, *n (%)*	​	​	0.36
Current smoker	15 (16%)^d^	26 (12%)^d^	​
Past smoker	20 (21%)	40 (18%)	​
Never smoked	59 (63%)	159 (71%)	​
Heavy alcohol consumption, *n (%)*	6 (7%)	6 (3%)	0.11
Ethnicity, *n (%)*	​	​	0.03
South-East/North-East Asian	26 (27%)	90 (40%)	​
Southern/Central Asian	2 (2%)	16 (7%)	​
European	56 (59%)	100 (44%)	​
Other^e^	11 (12%)	21 (9%)	​
Blood pressure (BP)^f^, *mean (SD)*			
Systolic BP	135.3 (14.2)	119.3 (12.2)	<0.01
Diastolic BP	76.9 (11.3)	69.7 (9.1)	<0.01
Number of antihypertensives, *n (%)*	​	​	<0.01
0	42 (44%)	157 (69%)	​
1	24 (25%)	50 (22%)	​
2	18 (19%)	16 (7%)	​
3	7 (7%)	4 (2%)	​
4 or greater	4 (4%)	0 (0%)	​
Antihypertensive medications, *n (%)*			
ACEi/ARB	46 (48%)	51 (22%)	<0.01
Beta blocker	17 (18%)	13 (6%)	<0.01
CCB	18 (19%)	20 (9%)	0.01
Diuretic	8 (8%)	4 (2%)	<0.01
MRA	4 (4%)	3 (1%)	0.20
Other	5 (5%)	1 (1%)	<0.01
Past history of comorbidities, *n (%)*			
Type 2 diabetes mellitus	4 (4%)	7 (3%)	0.74
Ischaemic heart disease	9 (9%)	8 (4%)	0.05
Heart failure	4 (4%)	3 (1%)	0.20
Obstructive sleep apnoea	4 (4%)	0 (0%)	<0.01
Stroke	17 (18%)	11 (5%)	<0.01
Chronic kidney disease	12 (13%)	5 (2%)	<0.01

ACEi: Angiotensin-converting enzyme inhibitor; ARB: Angiotensin II receptor blocker; CCB: calcium channel blocker; MRA: mineralocorticoid receptor antagonist.

^a^Age was normally distributed for the group with an indication for PA testing, with mean (SD) age: 47.8 (14.9) years.

^b^9 missing data.

^c^19 missing data.

^d^≤ 5 missing data.

^e^Includes the following ethnicities: Aboriginal and Torres Strait Islander, Pacific Islander, Latin American, African, Middle Eastern, other Asian ethnicities, and mixed ethnicities.

^f^Derived as the mean over the first three recorded blood pressure readings.

**Table 2.** Baseline SLE disease characteristics in patients by indication for PA testing.

Disease Characteristics	PA testing		*p*-value
	Indication (*n* = 95)	No indication (*n* = 227)	
Disease duration (years)*, median (IQR)*	8.5 (3.5–18.1)	5.6 (1.0–13.3)	<0.01
Disease activity (SLEDAI-2K)			
Active disease, *n (%)*	43 (45%)	91 (40%)	0.46
Highly active disease, *n (%)*	14 (15%)	22 (10%)	0.24
Active renal disease, *n (%)*	23 (24%)	41 (18%)	0.22
Flare (SFI)			
Mild-moderate flare, *n (%)*	14 (15%)	37 (16%)	0.87
Severe flare, *n (%)*	8 (8%)	17 (7%)	0.82
Any flare, *n (%)*	22 (23%)	54 (24%)	0.99
Organ damage (SDI)			
Overall organ damage, *median (IQR)*	1 (0–2)	0 (0–1)	<0.01
Overall damage, *n (%)*	63 (66%)	94 (41%)	<0.01
Renal damage, *n (%)*	17 (18%)	12 (5%)	<0.01
Cardiovascular damage, *n (%)*	14 (15%)	14 (6%)	0.01
Proteinuria^a^, *n (%)*	20 (21%)	36 (16%)^b^	0.26
eGFR <60 *(ml/min), n (%)*	21 (22%)	8 (4%)	<0.01
CRP >3 *(mg/L), n (%)*	47 (49%)	66 (29%)	<0.01
ESR ≥ 25 *(mm/hr), n (%)*	36 (38%)	61 (27%)	0.06
Anti-Sm positive*, n (%)*	14 (15%)^b^	48 (22%)^b^	0.21
Anti-Ro positive*, n (%)*	41 (44%)^b^	126 (57%)^b^	0.04
Anti-La positive*, n (%)*	18 (19%)^b^	45 (21%)^c^	0.88
Rheumatoid factor positive, *n (%)*	30 (37%)^d^	67 (34%)^e^	0.68
Medications use^f^, *n (%)*			
Glucocorticoid	61 (64%)	136 (60%)	0.53
Glucocorticoid dose (mg), *median (IQR)*	5 (0–10)	5 (0–10)	0.40
Hydroxychloroquine	72 (76%)	191 (84%)	0.08
Immunosuppressant^g^	52 (55%)	126 (56%)	0.90

CRP: C-reactive protein; eGFR, estimated glomerular filtration rate; ESR: erythrocyte sedimentation rate; La: Sjogren’s syndrome related antigen B; LN: lupus nephritis; Ro, Sjogren’s syndrome related antigen A; SDI: Systemic Lupus International Collaborating Clinics/American College of Rheumatology (SLICC/ACR) Damage Index; SFI: Safety of Estrogens in Lupus National Assessment (SELENA) Flare Index (SFI); SLEDAI-2K: Systemic Lupus Erythematosus Disease Activity Index 2000; Sm: Smith.

^a^Proteinuria was defined as urine protein to creatinine ratio >0.05 g/mmol, using the SLEDAI-2K cut-off for normal range.

^b^≤ 5 missing data.

^c^8 missing data.

^d^13 missing data.

^e^28 missing data.

^f^Refers to current use of medications at time of visit where data were analysed.

^g^Includes: azathioprine, cyclosporine, cyclophosphamide, leflunomide, mercaptopurine, methotrexate, mycophenolate, sulfasalazine, tacrolimus, abatacept, adalimumab, baracitinib, belimumab, certolizumab, etanercept, infliximab, rituximab, tocilizumab, tofacitnib.

We next sought to assess differences in disease outcomes between the two groups, using data collected longitudinally from the baseline visit to the final visit of the study period (Table 3). The median duration of follow-up was longer for the group with an indication for PA testing (7.7 [IQR 5.3–9.0] vs 6.4 [IQR 4.0–8.8] years; *p* = 0.03). Significantly more patients in the group with an indication for PA testing had accrual of overall organ damage compared with the group without indication (54% vs 22%; *p* < 0.01) with specifically cardiovascular (13% vs 4%; *p* < 0.01), and renal damage (14% vs 1%; *p* < 0.01). The group with an indication for PA testing also had a significantly higher proportion of patients who had proteinuria on at least one occasion during the study period (58% vs 33%; *p* < 0.01). Furthermore, there were significant differences between indicated and non-indicated groups for PA testing in annual flare rate (0.60 [IQR 0.27–1.08] vs 0.40 [IQR 0.12–0.82], respectively; *p* < 0.01), which remained significant when classifying flares as of mild-moderate or severe severity. While there was no significant difference in median disease activity across the study period as assessed using the adjusted mean SLEDAI-2K, a higher proportion of patients from the group with indications for PA testing attained High Disease Activity Status during the study period (54% vs 35%; *p* < 0.01). The group with an indication for PA testing also used higher doses of glucocorticoids over the follow-up period both cumulatively (7521 [IQR 2221–16198] vs 2490 [IQR 0–8989] mg, respectively*; p* < 0.01) and on average adjusted for irregular time intervals between visits (3.6 [IQR 0.8–6.6] vs 1.8 [IQR 0–4.9] mg, respectively*; p* < 0.01).

**Table 3.** SLE disease characteristics over time in patients by indication for PA testing.

*Disease Characteristics*	PA testing		*p*-value
	Indication (*n* = 95)	No indication (*n* = 227)	
Period of follow-up (years), *median (IQR)*	7.7 (5.3–9.0)	6.4 (4.0–8.8)	0.03
Disease duration at last visit (years), *median (IQR)*	16.1 (10.8–24.2)	12.3 (7.1–20.2)	<0.01
Disease activity over time (SLEDAI-2K)			
Adjusted mean SLEDAI-2K, *median (IQR)*	3.8 (2.0–5.3)	3.7 (2.2–4.8)	0.50
Adjusted mean SLEDAI-2K >4, *n (%)*	42 (44%)	92 (41%)	0.62
Highly active disease (ever), *n (%)*	51 (54%)	79 (35%)	<0.01
Proteinuria (ever)^a^, *n (%)*	55 (58%)	76 (34%)^b^	<0.01
Biopsy-confirmed LN, *n (%)*			
Class II	3 (3%)	0 (0%)	​
Class II and V	1 (1%)	6 (3%)	​
Class III	3 (3%)	4 (2%)	​
Class III and V	3 (3%)	7 (3%)	​
Class IV	9 (9%)	4 (2%)	​
Class IV and V	4 (4%)	8 (4%)	​
Class V	3 (3%)	5 (2%)	​
Class VI	1 (1%)	0 (0%)	​
Glucocorticoid use over time			
Cumulative prednisolone (mg), *median (IQR)*	7521 (2221–16198)	2490 (0–8989)	<0.01
Adjusted mean prednisolone (mg), *median (IQR)*	3.6 (0.8–6.6)	1.8 (0–4.9)	<0.01
Flare (ever) (SFI)			
Mild-moderate flare, *n (%)*	86 (91%)	171 (75%)	<0.01
Severe flare, *n (%)*	51 (54%)	77 (34%)	<0.01
Any flare, *n (%)*	88 (93%)	175 (77%)	<0.01
Annual average flare rate (SFI)			
Mild-moderate flare, *median (IQR)*	0.53 (0.22–0.86)	0.34 (0.11–0.67)	<0.01
Severe flare, *median (IQR)*	0.11 (0–0.33)	0 (0–0.15)	<0.01
Any flare, *median (IQR)*	0.60 (0.27–1.08)	0.40 (0.12–0.82)	<0.01
Organ damage at last recorded visit (SDI)			
Overall organ damage, *median (IQR)*	2 (1–4)	0 (0–1)	<0.01
Overall damage, *n (%)*	78 (82%)	114 (50%)	<0.01
Renal damage, *n (%)*	26 (27%)	14 (6%)	<0.01
Cardiovascular damage, *n (%)*	24 (25%)	21 (9%)	0.01
Accrual of organ damage (SDI)			
Overall organ damage, *n (%)*	51 (54%)	49 (22%)	<0.01
Renal damage, *n (%)*	13 (14%)	2 (1%)	<0.01
Cardiovascular damage, *n (%)*	12 (13%)	9 (4%)	<0.01

LN: lupus nephritis; SDI: Systemic Lupus International Collaborating Clinics/American College of Rheumatology (SLICC/ACR) Damage Index; SFI: Safety of Estrogens in Lupus National Assessment (SELENA) Flare Index (SFI); SLEDAI-2K: Systemic Lupus Erythematosus Disease Activity Index 2000.

^a^Proteinuria was defined as urine protein to creatinine ratio >0.05 g/mmol, using the SLEDAI-2K cut-off for normal range.

^b^≤ 5 missing data.

We then explored the relationships between having an indication for PA testing with SLE phenotypes and outcomes in the subset of patients with HTN (*n* = 178), using univariable and multivariable regression modelling. Having an indication for PA testing was significantly associated with reduced eGFR at baseline in univariable analysis (OR 3.64, 95% CI 1.47–10.38, *p* = 0.01) (Supplemental Table 1); this association remained significant after adjusting for age (adjusted OR 3.29, 95% CI 1.30–9.47, *p* = 0.02) (Table 4). Having an indication for PA testing was also significantly associated in univariable analysis with higher organ damage both as a continuous variable (IRR 1.50, 95% CI 1.05–2.14, *p* = 0.03) and binarized variable (OR 1.92, 95% CI 1.05–3.54, *p* = 0.03) (Supplemental Table 1). These associations remained significant after adjusting for age, ethnicity and disease duration for organ damage as a continuous variable (adjusted IRR 1.48, 95% CI 1.05–2.09, *p* = 0.03) (Table 4) and after adjusting for age and disease duration for organ damage as a binarized variable (adjusted OR 1.94, 95% CI 1.02–2.09, *p* = 0.04) (Table 4). Having an indication for PA testing was, however, not significantly associated with baseline cardiovascular or renal organ damage, disease activity or flare in multivariable analysis (Table 4).

**Table 4.** Multivariable associations between indication for PA testing and baseline SLE disease characteristics in the subset of hypertensive SLE patients.

Regression model		Adjusted odds ratio^a^	95% CI	*p*-value
Outcome	Exposure			
Active disease				
​	**PA indicated**	1.24	0.67, 2.31	0.50
​	Age	0.97	0.95, 0.99	0.01
​	Glucocorticoid use	1.56	0.83, 2.96	0.17
Highly active disease				
​	**PA indicated**	1.93	0.74, 5.37	0.19
​	Age	0.97	0.94, 1.01	0.13
​	Asian ethnicity	2.85	1.08, 7.97	0.04
​	Ethnicity (other)	0.82	0.04, 5.43	0.86
​	Glucocorticoid use	4.40	1.38, 19.65	0.02
Active renal disease				
​	**PA indicated**	1.25	0.60, 2.64	0.56
​	Age	0.97	0.94, 1.00	0.04
​	Asian ethnicity	1.73	0.81, 3.72	0.15
​	Ethnicity (other)	0.74	0.10, 3.30	0.72
​	Glucocorticoid use	1.54	0.66, 3.75	0.33
​	Immunosuppressant use	1.85	0.79, 4.52	0.17
Mild-moderate flare				
​	**PA indicated**	0.62	0.28, 1.34	0.23
Severe flare				
​	**PA indicated**	0.90	0.30, 2.75	0.86
​	Glucocorticoid use	10.10	1.95, 185.2	0.03
Any flare				
​	**PA indicated**	0.84	0.41, 1.70	0.63
Organ damage (continuous)^b^				
​	**PA indicated**	**1.48**	**1.05, 2.09**	**0.03**
​	Age	1.00	0.99, 1.02	0.57
​	Asian ethnicity	1.08	0.58, 2.03	0.35
​	Ethnicity (other)	0.84	0.58, 1.22	0.80
​	Disease duration	1.04	1.02, 1.05	<0.01
Organ damage (binary)				
​	**PA indicated**	**1.94**	**1.03, 3.73**	**0.04**
​	Age	1.02	0.99, 1.04	0.19
​	Disease duration	1.07	1.03, 1.12	<0.01
Renal damage				
​	**PA indicated**	2.08	0.84, 5.53	0.13
​	Asian ethnicity	1.67	0.64, 4.42	0.29
​	Ethnicity (other)	3.58	0.82, 14.2	0.07
​	Glucocorticoid use	1.43	0.51, 4.45	0.51
​	Immunosuppressant use	2.48	0.89, 7.78	0.10
Cardiovascular damage				
​	**PA indicated**	1.46	0.54, 4.20	0.46
​	Male sex	0.18	0.03, 1.15	0.11
​	Asian ethnicity	0.18	0.03, 0.67	0.03
​	Ethnicity (other)	1.72	0.34, 6.96	0.47
​	Disease duration	1.03	0.98, 1.07	0.23
Proteinuria				
​	**PA indicated**	1.07	0.51, 2.25	0.86
​	Age	0.97	0.95, 1.00	0.04
​	Asian ethnicity	1.68	0.79, 3.58	0.18
​	Ethnicity (other)	0.37	0.06, 2.55	0.37
​	Immunosuppressant use	2.25	1.00, 5.05	0.06
eGFR < 60 (ml/min/m^2^)				
​	**PA indicated**	**3.29**	**1.30, 9.47**	**0.02**
​	Age	1.04	1.01, 1.07	0.02
CRP >3 (mg/L)				
​	**PA indicated**	1.56	0.85, 2.87	0.15
​	Age	1.02	1.00, 1.04	0.10
ESR ≥25 (mm/hr)				
​	**PA indicated**	1.82	0.94, 3.59	0.08
​	Asian ethnicity	2.88	1.48, 5.73	<0.01
​	Ethnicity (other)	0.47	0.07, 1.93	0.35

95% CI: 95% confidence interval; CRP: C-reactive protein; eGFR: estimated glomerular filtration rate; ESR: erythrocyte sedimentation rate; PA: primary aldosteronism.

^a^Model used was logistic regression with effect size reported as adjusted Odds Ratio unless specified otherwise.

^b^A negative binomial regression model was fitted for skewed and over-dispersed count outcome data. Effect size reported as adjusted Incidence Rate Ratio.

When exploring longitudinal clinical data we found that having an indication for PA testing was significantly associated with a higher prevalence of flare of any type in univariable analysis (OR 3.24, 95% CI 1.32–8.79, *p* = 0.014) (Supplemental Table 2) and after adjustment for ethnicity and cumulative prednisolone usage (adjusted OR 2.86, 95% CI 1.04–8.60, *p* = 0.049) (Table 5). Of note, having an indication for PA testing was significantly associated in univariable analysis with proteinuria (OR 1.89, 95% CI 1.04–3.44, *p* = 0.04) and adjusted mean prednisolone (OR 1.75, 95% CI 1.05–2.91, *p* = 0.03), as well as prevalence of mild-moderate flare (OR 2.65, 95% CI 1.14–6.53, *p* = 0.03), and both prevalence (OR 2.05, 95% CI 1.13–3.77, p = 0.02) and annual average rate of severe flare (OR 1.41, 95% CI 1.09–1.82, *p* = 0.01) (Supplemental Table 2), but these associations did not remain significant in multivariable analysis (Table 5).

**Table 5.** Multivariable associations between indication for PA testing and SLE disease characteristics over time in the subset of hypertensive SLE patients.

Regression model Outcome		Effect size^a^	95% CI	*p*-value
​	Exposure			
Adjusted mean SLEDAI-2K				
*Linear Regression*	**PA indicated**	0.07	−0.59, 0.73	0.84
	Age	−0.05	−0.07, −0.02	<0.01
	Asian ethnicity	0.69	0.02, 1.36	0.05
	Ethnicity (other)	0.34	−0.09, 1.55	0.59
	Cum. Prednisolone^b^	0.10	0.06, 0.13	<0.01
Adjusted mean SLEDAI-2K >4				
*Logistic Regression*	**PA indicated**	0.75	0.37, 1.50	0.41
	Age	0.96	0.93, 0.98	<0.01
	Cum. Prednisolone^b^	1.09	1.05, 1.14	<0.01
Highly active disease (ever)				
*Logistic Regression*	**PA indicated**	1.74	0.88, 3.50	0.11
	Age	0.98	0.95, 1.00	0.07
	Asian ethnicity	1.69	0.85, 3.40	0.14
	Ethnicity (other)	0.48	0.11, 1.83	0.31
	Cum. Prednisolone^b^	1.08	1.04, 1.13	<0.01
Proteinuria (ever)				
*Logistic Regression*	**PA indicated**	1.95	0.99, 3.91	0.06
	Age	0.97	0.95, 1.00	0.02
	Asian ethnicity	2.02	1.01, 4.09	0.5
	Ethnicity (other)	2.34	0.65, 8.97	0.20
	Cum. Prednisolone^b^	1.06	1.02, 1.11	<0.01
Adjusted mean prednisolone				
*Log-linear Regression*	**PA indicated**	1.29	0.99, 1.69	0.06
Mild-moderate flare				
*Logistic Regression*	**PA indicated**	2.25	0.88, 6.08	0.10
	Asian ethnicity	2.51	0.89, 7.99	0.10
	Ethnicity (other)	1.49	0.28, 11.6	0.66
	Cum. Prednisolone^b^	1.28	1.13, 1.50	<0.01
Severe flare				
*Logistic Regression*	**PA indicated**	1.48	0.76, 2.89	0.25
	Cum. Prednisolone^b^	1.13	1.08, 1.19	<0.01
Any flare				
*Logistic Regression*	**PA indicated**	**2.86**	**1.04, 8.60**	**0.05**
	Asian ethnicity	2.93	0.96, 10.5	0.07
	Ethnicity (other)	1.39	0.25, 11.2	0.72
	Cum. Prednisolone^b^	1.35	1.16, 1.67	<0.01
Annual average flare – mild-moderate flare				
*Log-linear Regression*	**PA indicated**	1.20	0.75, 1.92	0.44
	Age	0.99	0.98, 1.01	0.47
	Asian ethnicity	1.35	0.84, 2.17	0.22
	Ethnicity (other)	0.73	0.31, 1.74	0.47
	Cum. Prednisolone^b^	1.05	1.03, 1.08	<0.01
Annual average flare – severe flare				
*Log-linear Regression*	**PA indicated**	1.20	0.94, 1.53	0.13
	Age	0.99	0.99, 1.00	0.22
	Asian ethnicity	0.97	0.76, 1.24	0.82
	Ethnicity (other)	0.80	0.51, 1.24	0.32
	Cum. Prednisolone^b^	1.04	1.03, 1.05	<0.01
Annual average flare – any flare				
*Log-linear regression*	**PA indicated**	1.10	0.83, 1.45	0.52
	Age	0.99	0.98, 1.00	0.13
	Asian ethnicity	1.10	0.83, 1.46	0.51
	Ethnicity (other)	0.73	0.43, 1.23	0.24
	Cum. Prednisolone^b^	1.04	1.03, 1.05	<0.01
Overall organ damage at last visit				
*Logistic Regression*	**PA indicated**	0.81	0.42, 1.54	0.52
	Cum. Prednisolone^b^	1.02	0.99, 1.06	0.20
	Baseline organ damage	2.34	1.24, 4.48	0.01
Renal damage at last visit				
*Logistic Regression*	**PA indicated**	1.72	0.67, 4.79	0.27
Cardiovascular damage at last visit				
*Logistic Regression*	**PA indicated**	0.89	0.37, 2.10	0.79
	Male sex	0.15	0.02, 1.14	0.07
	Baseline cardiovascular damage	2.33	0.79, 6.85	0.13
Accrual organ damage				
*Logistic Regression*	**PA indicated**	1.43	0.75, 2.76	0.28
	Age	1.02	0.99, 1.04	0.21
	Disease duration	1.02	0.98, 1.05	0.32
	Baseline organ damage	1.54	0.77, 3.14	0.23
Accrual renal damage				
*Logistic Regression*	**PA indicated**	0.85	0.17, 4.66	0.85
	Age	1.06	1.00, 1.13	0.06
Accrual cardiovascular damage				
*Logistic Regression*	**PA indicated**	1.20	0.36, 4.31	0.77
	Age	1.02	0.98, 1.07	0.28
	Disease duration	1.05	1.00, 1.11	0.05

95% CI: 95% confidence interval; PA: primary aldosteronism; SLEDAI-2K: Systemic Lupus Erythematosus Disease Activity Index 2000.

^a^Effect size reported as estimated coefficient (β) for linear regression, and adjusted odds ratio for logistic regression. For log-transformed linear regression, the effect size is presented by exponentiating the coefficients (e^β^) to represent the ratio of odds, making them interpretable as relative risks.

^b^Cumulative Prednisolone divided by 1000 due to large values in order to provide reasonable effect size.

## Discussion

In this study, we found that patients with SLE with indications for PA testing were significantly different from those without, in terms of baseline characteristics. Some differences were expected, such as an older age, concurrent obstructive sleep apnoea and higher BMI in the group with indications for PA testing, as these are well established risk factors for hypertension.^27^ For other observed differences, it is unclear whether they may relate to SLE rather than potential underlying PA. Differences in baseline renal and cardiovascular damage, and accrual of both renal and cardiovascular damage over the study period, were seen in patients with an indication for PA testing compared to those without. Some of these differences may be unsurprising in the subgroup meeting criteria for PA testing as such outcomes are known to be affected by blood pressure and only differences in baseline organ damage, baseline renal function, and prevalence of flare of any type over the study period remained significant after adjustment for relevant covariates in a multivariable analysis of a subset consisting only of patients with HTN. However, aldosterone importantly is also known to cause additional end organ damage independent of hypertension, and having an indication for PA testing remained significantly associated with higher organ damage at baseline in the multivariable analysis, even compared to other hypertensive patients without an indication, after adjusting for age and disease duration.

Having an indication for PA testing was significantly associated with reduced renal function at baseline, in multivariable analysis compared with other hypertensive patients. Both uncontrolled hypertension and aldosterone excess per se are known to cause renal damage^28,29^; whether this reflects a possible contribution from undiagnosed PA remains to be elucidated. A significant association was also observed between an indication for PA testing and the prevalence of flare over the study period after adjustment for ethnicity and cumulative prednisolone usage, suggesting a more severe phenotype. Notably, however, we did not observe an association between having an indication for PA testing and renal organ damage either at baseline or accrued over time. It is recognized that in patients with hyperaldosteronism, aldosterone-induced glomerular hyperfiltration may increase the eGFR thereby masking underlying chronic disease.^30^ Nevertheless, the ascertainment of a contribution of PA to renal disease and hypertension severity in patients with SLE will require a prospective study, as few of the patients in this study had actually been investigated for PA, and none had a confirmed diagnosis.^16^

A key limitation is that the exposure variable in this study was the presence of an indication for PA testing rather than confirmed PA. As these indications include clinical features associated with more severe hypertension and comorbidity, the observed associations may reflect confounding by indication rather than an effect of aldosterone excess. In addition, the multivariable models were limited by sample size and the number of covariates that could be included, and residual confounding cannot be excluded. A further caveat of this study is that no data on salt intake or the dietary profile of the patients were available in the ALRB, nor were data on secondary antiphospholipid syndrome able to be reported. Use of hydroxychloroquine in this cohort, which is thought to reduce cardiovascular risk and is generally recommended for all patients with SLE unless contraindicated, was in line with other cohort studies, where its reported rates of use ranged between 50% to over 75%.^31,32,33,34,35,36^ The prevalence of rheumatoid factor positivity in this cohort on the other hand was slightly higher than the prevalence of 20-30% generally reported in other cohorts,^37,38^ although this may be related to the presence of missing data in 12.7 % (41/322) of the cohort.

In conclusion, we found that renal impairment, organ damage, as well as prevalence of flare over the study period, were more pronounced in patients with an indication for PA testing compared to those with hypertension without an indication. It remains, however, unclear how much of the renal and cardiovascular disease burden in SLE could be potentially attributed to PA and/or other factors such as direct renal injury from lupus nephritis, SLE related vasculopathy, or long-term exposure to glucocorticoids and other agents such as NSAIDs. Nonetheless, our findings highlight that hypertension in SLE is an important yet not fully understood and sub-optimally managed issue in some patients. In addition to SLE-related factors, it is prudent to consider secondary causes of hypertension including PA which is common, highly treatable and potentially curable, but also others such as renal artery stenosis and obstructive sleep apnoea. A prospective study specifically designed to assess the prevalence and clinical impact of PA in patients with SLE is warranted to better understand its burden of disease in this high-risk population.

## Supplemental material

Supplemental Material - Clinical phenotypes and disease outcomes of patients with systemic lupus erythematosus meeting indication for primary aldosteronism testing Supplemental Material for Clinical phenotypes and disease outcomes of patients with systemic lupus erythematosus meeting indication for primary aldosteronism testing by Matthew Wong, Muhammad Akram, Joanna R. Kent, Peter J. Fuller, Alberta Y. Hoi, Jun Yang and Fabien B. Vincent in Lupus

Supplemental Material - Clinical phenotypes and disease outcomes of patients with systemic lupus erythematosus meeting indication for primary aldosteronism testing Supplemental Material for Clinical phenotypes and disease outcomes of patients with systemic lupus erythematosus meeting indication for primary aldosteronism testing by Matthew Wong, Muhammad Akram, Joanna R. Kent, Peter J. Fuller, Alberta Y. Hoi, Jun Yang and Fabien B. Vincent in Lupus

## Data Availability

All data relevant to the study are included in this article.*
