# Supplementary material for: Clinical phenotypes and disease outcomes of patients with systemic lupus erythematosus meeting indication for primary aldosteronism testing
Source: Lupus. 2026 Jul 31;35(12):1308–19. doi: 10.1177/09612033261473968 (PMC13569909; doi:10.1177/09612033261473968)
Supplement: Supplementary file 1 [file sj-pdf-1-lup-10.1177_09612033261473968.pdf]

Supplementary Table 1. Univariable associations between having an indication for PA testing and baseline SLE disease characteristics in the subset of hypertensive SLE patients.

Regression Model	Effect Size[1]	95% CI	*p*-value
**Active Disease** ~ PA Indicated	1.13	0.63, 2.06	0.68
**Highly Active Disease** ~ PA Indicated	1.62	0.66, 4.26	0.31
**Active Renal Disease** ~ PA Indicated	1.08	0.54, 2.17	0.84
**Mild-moderate flare** ~ PA Indicated	0.62	0.28, 1.34	0.23
**Severe Flare** ~ PA Indicated	1.00	0.34, 2.97	1.00
**Any Flare** ~ PA Indicated	0.84	0.41, 1.70	0.63
**Organ Damage (continuous)** ~ PA Indicated[2]	**1.50**	**1.05, 2.14**	**0.03**
**Organ Damage (binary)** ~ PA Indicated	**1.92**	**1.05, 3.54**	**0.03**
**Renal Damage** ~ PA Indicated	2.04	0.85, 5.27	0.12
**Cardiovascular Damage** ~ PA Indicated	1.88	0.74, 5.18	0.20
**Proteinuria** ~ PA Indicated	0.90	0.44, 1.84	0.77
**eGFR <60 (ml/min/1.73m^2)** ~ PA Indicated	**3.64**	**1.47, 10.38**	**< 0.01**
**CRP >3 (mg/L)** ~ PA Indicated	1.64	0.90, 3.01	0.11
**ESR ≥25 (mm/hr)** ~ PA Indicated	1.50	0.80, 2.84	0.21

[1] Model used was logistic regression with effect size reported as Odds Ratio unless specified otherwise.

[2] A negative binomial regression model was fitted for skewed and over-dispersed count outcome data. Effect size reported as adjusted Incidence Rate Ratio.

95% CI, 95% confidence interval; CRP, C-reactive protein; eGFR, estimated glomerular filtration rate; ESR, erythrocyte sedimentation rate; PA: primary aldosteronism
