# Supplementary material for: Clinical phenotypes and disease outcomes of patients with systemic lupus erythematosus meeting indication for primary aldosteronism testing
Source: Lupus. 2026 Jul 31;35(12):1308–19. doi: 10.1177/09612033261473968 (PMC13569909; doi:10.1177/09612033261473968)
Supplement: Supplementary file 2 [file sj-pdf-2-lup-10.1177_09612033261473968.pdf]

Supplementary Table 2. Univariable associations between having an indication for PA testing and SLE disease characteristics over time in the subset of hypertensive SLE patients.

Regression Model Outcome	Model Type	Effect Size[1]	95% CI	*p*-value
**Adjusted Mean SLEDAI-2K** ~ PA Indicated	Linear	β 0.20	-0.53, 0.94	0.59
**Adjusted Mean SLEDAI-2K > 4** ~ PA Indicated	Logistic	OR 0.89	0.49, 1.62	0.71
**Highly Active Disease (Ever)** ~ PA Indicated	Logistic	OR 1.76	0.97, 3.21	0.06
**Proteinuria** ~ PA Indicated	Logistic	**OR 1.89**	**1.04, 3.44**	**0.04**
**Adjusted mean prednisolone** ~ PA Indicated	Log Linear	**1.75**	**1.05, 2.91**	**0.03**
**Mild-moderate flare** ~ PA Indicated	Logistic	**OR 2.65**	**1.14, 6.53**	**0.03**
**Severe flare** ~ PA Indicated	Logistic	**OR 2.05**	**1.13, 3.77**	**0.02**
**Any flare** ~ PA Indicated	Logistic	**OR 3.24**	**1.32, 8.79**	**0.01**
**Annual average mild-moderate flare** ~ PA Indicated	Log Linear	1.11	0.85, 1.45	0.44
**Annual average severe flare** ~ PA Indicated	Log Linear	**1.41**	**1.09, 1.82**	**0.01**
**Annual average any flare** ~ PA Indicated	Log Linear	1.23	0.92, 1.65	0.17
**Overall damage at last visit** ~ PA Indicated	Logistic	1.02	0.55, 1.88	0.94
**Renal damage at last visit** ~ PA Indicated	Logistic	1.72	0.67, 4.79	0.27
**Cardiovascular damage at last visit** ~ PA Indicated	Logistic	1.02	0.44, 2.39	0.96
**Accrual of organ damage** ~ PA Indicated	Logistic	1.64	0.88, 3.10	0.12
**Accrual of renal damage** ~ PA Indicated	Logistic	1.17	0.25, 6.10	0.84
**Accrual of cardiovascular damage** ~ PA Indicated	Logistic	1.43	0.46, 4.92	0.54

[1]Effect size reported as estimated coefficient (β) for linear regression, odds ratio for logistic regression. For log-transformed linear regression, the effect size is presented by exponentiating the coefficients (e^{β}) to represent the ratio of odds, making them interpretable as relative risks.

95% CI, 95% confidence interval; SLEDAI-2K, Systemic Lupus Erythematosus Disease Activity Index 2000.
